# Homicide in Canada and the crime drop

**DOI:** 10.1186/s40163-017-0076-y

**Published:** 2018-01-24

**Authors:** Graham Farrell, Tarah Hodgkinson, Martin A. Andresen

**Affiliations:** 1 Centre for Criminal Justice Studies, School of Law, University of Leeds, Leeds, LS2 9JT UK; 2 Institute for Canadian Urban Research Studies and School of Criminology, Simon Fraser University, 8888 University Drive, Burnaby, BC V5A 1S6 Canada

**Keywords:** Homicide, Crime in Canada, Homicide in Canada, Crime decline, Crime drop, Crime falls, Security hypothesis, Debut crime hypothesis

## Abstract

In contrast to the Canadian crime drop of the 1990s, homicide appeared as an anomaly with a peak in the 1970s. Yet previous studies tend to refer only to completed homicides, and here we also include attempts. The resulting trend is remarkably similar to that in Canadian property crime for five decades. This seems unlikely to be a coincidence and we speculate about a causal link.

## Introduction

The existence of a Canadian crime drop is well established (Ouimet 1999, 2002; Pottie Bunge et al. 2005; Mishra and Lalumiere 2009; Farrell and Brantingham 2013; Hodgkinson et al. 2016) and similar to that of other high income countries (Zimring 2006; Tseloni et al. 2010). The homicide rate in Canada, however, has long appeared to be an anomaly, declining from the 1970s. The potential anomaly is important because it implies the drivers of homicide trends differ from those of other types of crime.

Here we suggest that the apparently anomalous homicide trend is an artefact of the definition of homicide that has been utilised which included only completed homicides. We combine attempted and completed homicides to produce an aggregate trend, for the following reasons. In Canadian law most attempted and completed crimes are aggregated—an attempted robbery is considered as a robbery for example—and the only form of crime that is differentiated is homicide. In addition, it could be argued that since intent is often the same for attempted and completed homicides, their aggregation is preferable when the cause of *behavioural* change is sought. Other definitional issues relating to the distinction between serious assaults and attempted homicide are discussed in the literature (see e.g. Harris et al. 2002; Andresen 2007; Cook et al. 2017), but we suggest there is a strong case for adding attempts and completions together, particularly since the resulting trend is informative. The resulting trend is found to closely track that of property crime over the last 50 years, and in recent decades the aggregate homicide trend is consistent with that of the more general Canadian crime drop.

## Data and methods

The homicide data used here are from the Homicide Survey conducted by The Canadian Centre for Justice Statistics of Statistics Canada and detailed in the report *Homicide in Canada 2015* (Mulligan et al. 2016). Note that ‘attempted murder’ is the term used in official reports but for simplicity we use ‘attempted homicide’ here. We calculate total homicides as the sum of attempted and completed homicides.

The property crime rates used here are from the annual Uniform Crime Reporting Survey (UCR Survey) and detailed in the report *Police Reported Crime Statistics in Canada, 2016* (Keighley 2017). At the time of writing these are the most recently available sources.

Data from 1965 to 2015 are examined, which is the period common to both data sets. The use of published data is, we suggest, a strength of this study: it establishes the independence of the data from the authors and adds transparency by allowing interested readers to peruse the primary publications for further methodological specifics as required.

## Findings

**Fig. 1:**
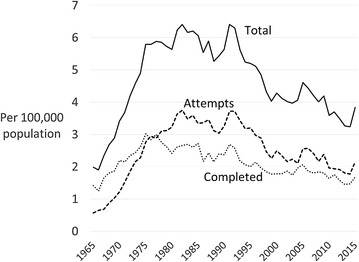
Homicide in Canada 1965–2015

From 1965 to 1975, both attempted and completed homicides increased rapidly (Fig. 1). In 1975, completed homicides peaked whereas attempts continued to increase until the early 1980s. The rate of attempted murders overtook that of completed homicides in 1976. Completed homicides trended downwards from 1975 with secondary peaks in 1986 and 1991. From 1982, attempts and completions largely track each other, trending downwards for several years then rising to a peak in 1991 whereafter they go into prolonged decline. It is clear that the main difference between the trends in completions and attempts is in the two decades from 1965 when the rate and duration of the increase in attempts was more extensive.

Total homicide increased steeply for two decades then began to level off from 1975 (Fig. 1). From 1975 the rate was mostly around 6 persons per 100,000 population but this began to fall steeply after the 1991 peak. From 1991 the rate declined for more than two decades and by 40.1% by 2015.

**Fig. 2:**
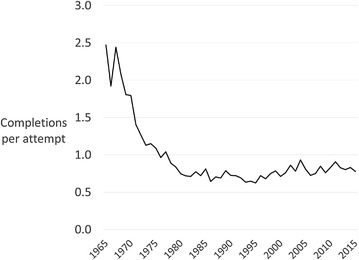
Completed homicides per attempted homicide in Canada 1965–2015

In the mid-1960s there were more than two completed homicides for every attempted homicide (Fig. 2). From the late 1960s this ratio fell steadily for around 15 years so that by 1982 there was only 0.7 completed homicides for every attempted homicide. From 1982 onwards the ratio of completed to attempted homicides remained stable, increasing slightly after 2000 but never exceeding 0.9 completions per attempt. That is, the likelihood of a homicide being completed rather than remaining an attempt falls before but not after 1982.

**Fig. 3:**
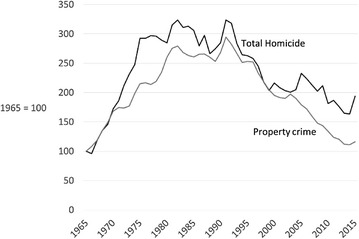
Trends in homicide (completed and attempts) and property crime, Canada 1965–2015

When the aggregate homicide trend is compared to the trend in Canadian property crime, the similarity is striking (Fig. 3). The strong correlation (r = 0.91) is such that it seems unlikely to be either coincidental or an artefact of method. Of course, correlation is distinct from causality, which is discussed next.

## Discussion

There is strong evidence from other high income countries including Australia, England and Wales, Germany, the Netherlands and the United States that the decline in property crimes in the 1990s was due to various security improvements (see e.g. Farrell 2013; Tilley et al. 2015; Tseloni et al. 2017). While further research is needed it seems reasonable to anticipate that security played a role in the Canadian property crime decline.

Building on the security hypothesis, the debut crime hypothesis proposes that adolescent offenders, unable to undertake the easy property crimes of adolescent onset, do not progress to violent crime (Farrell et al. 2015). Numerically, homicide is a tiny proportion of all crime and might be expected to decline as a component of a more general decline in violence. There is clear evidence that the crime decline occurred hugely disproportionately among young offenders. En passant, we note the potential for further research of these issues using the CANSIM data of Statistics Canada.

## Conclusion

When attempted and completed homicides are aggregated, the resulting trend is remarkably similar to that in property crime across five decades. With respect to the crime drop, the case could be made that both peaked in the early 1980s and that the 1991 peak is the outlier, but our main point here is that the two trends are the same. This leads to two conclusions: (1) Homicide should be considered a component of the more general Canadian crime drop and, (2) The security hypothesis and debut crime hypothesis should be considered by further research relating to Canadian homicide. It also raises the possibility that the United Kingdom's seemingly anomalous homicide trend might be an artefact, and that other homicide trends, such as that of the United States, might benefit from similar analysis.
